# Developing efficient strategies for localizing the enhanced yellow fluorescent protein subcellularly in transgenic *Eimeria* parasites

**DOI:** 10.1038/s41598-024-55569-6

**Published:** 2024-02-28

**Authors:** Ying Yu, Sixin Zhang, Chunhui Duan, Colin Crouch, Jingxia Suo, Xinming Tang, Xianyong Liu, Jie Liu, Beth Bruton, Ian Tarpey, Xun Suo

**Affiliations:** 1 National Key Laboratory of Veterinary Public Health and Safety, Key Laboratory of Animal Epidemiology and Zoonosis of Ministry of Agriculture, National Animal Protozoa Laboratory & College of Veterinary Medicine, China Agricultural University, Beijing, 100193 China; 2 MSD Animal Health, Walton Manor, Milton Keynes, MK7 7AJ UK; 3 Key Laboratory of Animal Biosafety Risk Prevention and Control (North) of MARA, Institute of Animal Sciences, Chinese Academy of Agricultural Sciences, Beijing, China

**Keywords:** Heterogenous antigen, Location, *Eimeria*, Surface, Microneme, Molecular biology, Parasitology

## Abstract

*Eimeria* species serve as promising eukaryotic vaccine vectors. And that the location of heterologous antigens in the subcellular components of genetically modified *Eimeria* may determine the magnitude and type of immune responses. Therefore, our study aimed to target a heterologous fluorescent protein to the cell surface or microneme, two locations where are more effective in inducing protective immunity, of *Eimeria tenella* and *E. acervulina* sporozoites. We used an enhanced yellow fluorescent protein (EYFP) as a tagging biomarker, fusing variously with some localization or whole sequences of compartmental proteins for targeting. After acquiring stable transgenic *Eimeria* populations, we observed EYFP expressing in expected locations with certain strategies. That is, EYFP successfully localized to the surface when it was fused between signal peptides and mature products of surface antigen 1 (SAG1). Furthermore, EYFP was efficiently targeted to the apical end, an optimal location for secretory organelle known as the microneme, when fused to the C terminus of microneme protein 2. Unexpectedly, EYFP exhibited dominantly in the apical end with only weak expression on the surface of the transgenic sporozoites when the parasites were transfected with plasmid with EYFP fused between signal peptides and mature products of *E. tenella* SAG 13. These strategies worked in both *E. tenella* and *E. acervulina*, laying a solid foundation for studying *E. tenella* and *E. acervulina*-based live vaccines that can be further tailored to the inclusion of cargo immunogens from other pathogens.

## Introduction

As single-celled and eukaryotic apicomplexan parasites, *Eimeria* spp. are frequently seen in the intestines of animals, especially in livestock and poultry with clinical coccidiosis^1,2^. Wild-type or attenuated *Eimeria* parasites have been used as effective vaccines against chicken coccidiosis^3–5^. Using a reverse genetics approach, *Eimeria* can also be converted to a vector for delivering foreign antigens as multivalent vaccines^6–10^. The cargo immunogens may be targeted into various subcellular compartments, including rhoptries, micronemes, dense granules, refractile bodies (RBs) and so on. In general, secreted or cell-surface antigens are more effective than their cytosolic counterparts in inducing protective immunity^11–13^. Thus, genetic modification with sequences that encode anchoring or targeting peptides is often preferred, as they ensure specific localization of heterogenous antigens to the desired subcellular sites^14^.

Like other apicomplexan parasites of medical and veterinary importance, *Eimeria* species use the surface antigen (SAG) with a C-terminal glycosylphosphatidylinositol (GPI)-anchored sequence to initially attach to its host cells^15,16^. Subsequently, the parasite uses its adhesive microneme protein (MIC), which is secreted by the anterior organelle microneme, to tighten attachment to host cells^17,18^. The invading parasite then injects its secretory rhoptry neck protein into the host cytoplasm to build moving junctions, and with the help of rhoptry protein (ROP), the invasion stage of each infection is completed^19,20^.

Early studies have shown that P30 is a major surface SAG protein of *Toxoplasma gondii*, and deletion of its GPI-anchored sequence results in a changed localization of fused green fluorescent protein (GFP) to the parasitophorous vacuole but not the membrane in stable transformants^21^. The *E. tenella* MIC-2 (EtMIC2) localizes to the cytoplasmic vesicles via its signal peptide (SP2); fusion of the GPI-anchor sequence of *E. tenella* SAG-1 (EtSAG1) to the SP2 C-terminus results in an altered localization to cell surface^13^. These observations have established an important role that GPI-anchor sequence plays in the cell membrane domain. Studies have also shown that the *T. gondii* ROP-1 (TgROP1) and the *T. gondii* MIC-3 (TgMIC3) could target fused GFP to the rhoptry or microneme, respectively, in *T. gondii*^22^. These targeting strategies could be considered to apply to develop effective vaccines with surface and/or microneme-localized antigens when genetically engineered *Eimeria* parasites are used as vectors.

*Eimeria tenella* is a model species of *Eimeria.* And *E. acervulina* causes chicken coccidiosis with a moderate pathogenesis; but interestingly, the infection results in a high level of host immunity. Thus, *E. acervulina* is also considered an adequate candidate for developing transgenic *Eimeria*-based vaccines^23^. In this study, we took various approaches for expressing heterologous antigens in two transgenic *Eimeria* organisms with surface-presented or microneme-localized antigens.

## Results

### The signal peptide and mature peptide from SAGs targeted the EYFP to *E. tenella* cell surface

**Figure 1:**
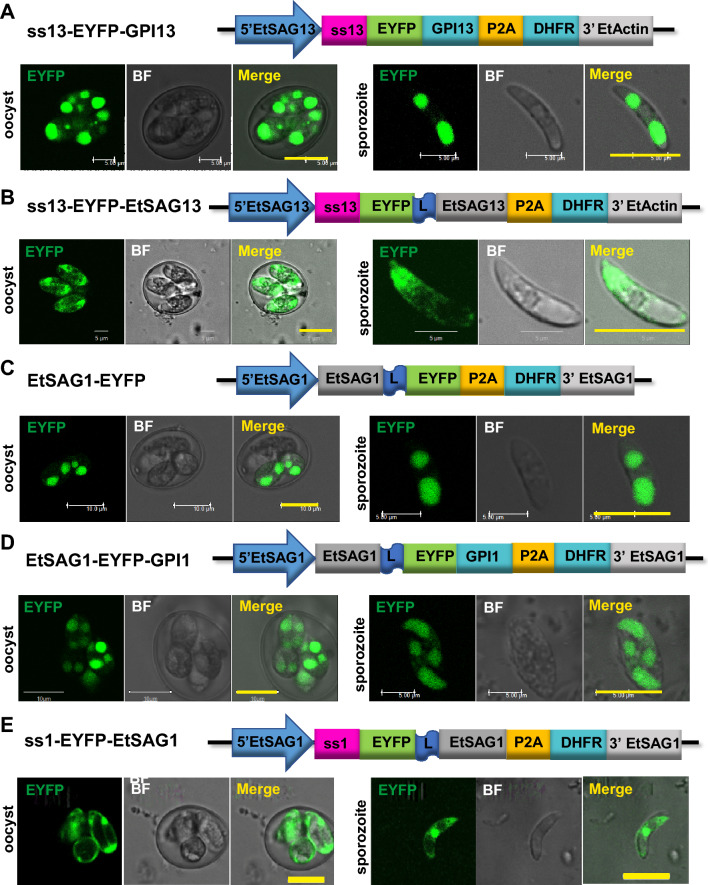
Confocal microscopy of EYFP expression in *E. tenella* transfected with various EtSAG13 or EtSAG1 constructs. (**A**) Construct ss13-EYFP-GPI13, in which EYFP was fused to the C-terminal of signal peptide from EtSAG13 (ss13), and to the N-terminal of GPI from EtSAG13 (GPI13): EYFP was expressed in refractile bodies (RBs). (**B**) Construct ss13-EYFP-EtSAG13, in which EYFP was inserted into EtSAG13 after its signal peptide: EYFP was expressed in microneme. (**C**) Construct EtSAG1-EYFP, in which EYFP was fused to the C-terminal of EtSAG1: EYFP was detected in RBs. (**D**) Construct EtSAG1-EYFP-GPI1, in which EYFP was inserted into EtSAG1 before its GPI-anchored protein (GPI1): EYFP was detected in RBs. (**E**) Construct ss1-EYFP-EtSAG1, in which EYFP was fused into EtSAG1 after its signal peptide (ss1): EYFP was present on cell surface and in nucleus. Bar = 10 μm. 5′EtSAG13, the upstream untranslated sequence from EtSAG13, as promoter; P2A^38^, a self-cleavage peptide of porcine teschovirus-1, was used to separate expression of flanking genes; DHFR, dihydrofolate reductase-thymidylate synthase mutants of *Toxoplasma gondii*, resistant to pyrimethamine, for drug selection of transgenic populations; 3′EtActin, the downstream untranslated sequence from *E. tenella* actin protein (EtActin); 5′EtSAG1, the upstream untranslated sequence from EtSAG1, as promoter; L, linker peptide, the sequence is GS(GGGS)_2_GS; 3′EtSAG1, the downstream untranslated sequence from EtSAG1; *BF* bright field.

EYFP was inserted between the signal peptide of EtSAG13 and the GPI–anchored sequence of EtSAG13 or the whole sequence minus the signal peptide, resulting in two constructs termed ss13-EYFP-GPI13 and ss13-EYFP-EtSAG13, respectively (Fig. 1A,B). In stably transfected parasites, i.e., sporulated oocysts, and released sporozoites, confocal fluorescent microscopy revealed that EYFP was expressed in RBs of transgenic sporozoites which were transfected with ss13-EYFP-GPI13 (Fig. 1A), but on transgenic sporozoites transfected with ss13-EYFP-EtSAG13, EYFP was unexpectedly expressed dominantly in apical end with only week expression on the surface of the parasite (Fig. 1B).

In another experiment, we fused EYFP in different sequence location of EtSAG1: EYFP was fused to the C-terminal of EtSAG1(EtSAG1-EYFP, Fig. 1C), or inserted into EtSAG1 before GPI-anchored protein (EtSAG1-EYFP-GPI, Fig. 1D), or inserted into EtSAG1 after signal peptide (ss- EYFP-EtSAG1, Fig. 1E). After stable transfection in *E. tenella*, constructs EtSAG1-EYFP-GPI and EtSAG1-EYFP had EYFP expression in the RBs of sporozoites (Fig. 1C,D). In contrast, construct ss-EYFP-EtSAG1 had EYFP on cell surface and in the nucleus of sporozoites (Fig. 1E).

### MIC2 of *E. tenella* (EtMIC2) or *E. acervulina* (EaMIC2) delivered target proteins to apical end of sporozoites

**Figure 2:**
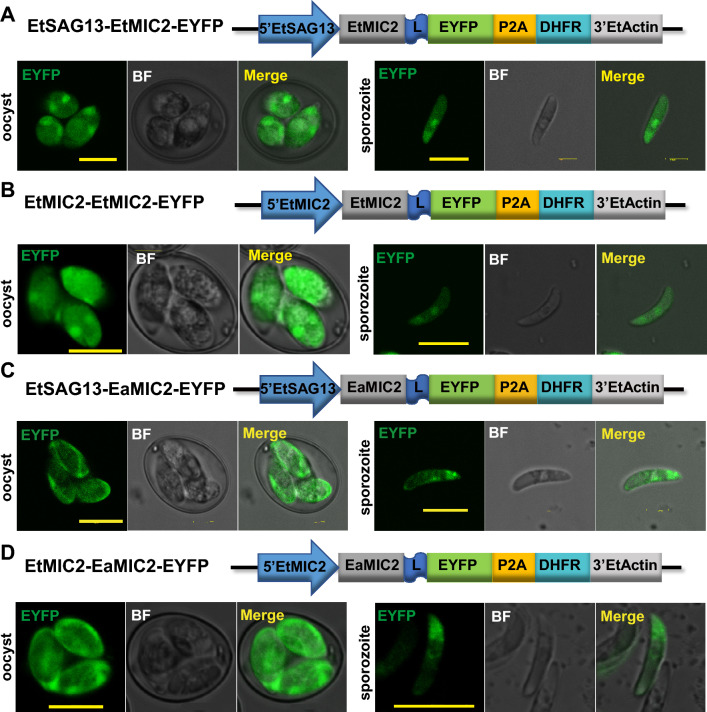
EYFP expression in *E. tenella* transfected with microneme protein 2 (MIC2)-related plasmids. (**A**) Plasmid EtSAG13-EtMIC2-EYFP, in which EYFP was fused to the C terminal of *E. tenella* MIC-2 (EtMIC2) under the regulation of promoter 5′EtSAG13. (**B**) Plasmid EtSAG13-EaMIC2-EYFP, in which EYFP was fused to *E. acervulina* MIC-2 (EaMIC2) under the regulation of 5′EtSAG13. (**C**) Plasmid EtMIC2-EtMIC2-EYFP, in which EYFP was fused to EtMIC2 under the regulation of EtMIC2 promoter (5′EtMIC2). (**D**) Plasmid EtMIC2-EaMIC2-EYFP, in which EYFP was fused to EaMIC2 under the regulation of 5′EtMIC2. In all experiments, EYFP was observed in micronemes alone or in both microneme and nucleus in sporozoites. Bar = 10 μm.

We designed constructs in which EYFP was fused behind the full coding sequence of EtMIC2 (Fig. 2A,B) or EaMIC2 (Fig. 2C,D), with both under the control of different promoters, i. e. the upstream untranslated sequence from EtSAG13 (5′EtSAG13, Fig. 2A,C) or EtMIC2 (5′ EtMIC2, Fig. 2B,D). When fused to the EtMIC2, EYFP was expressed in apical end and nucleus (Fig. 2A,B). When fused to the EaMIC2, EYFP accumulated mainly in micronemes (Fig. 2C,D).

### Genetic manipulation in *E. acervulina*

**Figure 3:**
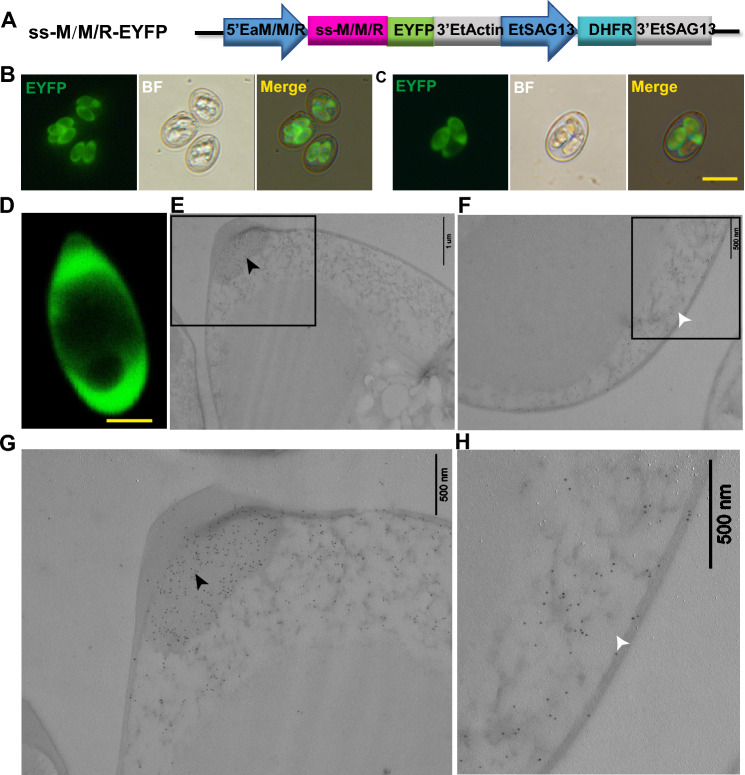
Detection of target proteins in *E. acervulina* transfected with various plasmid constructs. (**A**) The schematic of plasmids ss-M/M/R-EYFP, in which EYFP was fused to promoter and signal sequence from microneme adhesive repeat (MAR) domain containing protein 1 (MCP1), MIC, or ROP (M/M/R). (**B**,**C**) EYFP expression controlled by promoter and signal sequence of EaMCP1 (**B**) or EaMIC2 (**C**) in sporulated oocysts. Bar = 20 μm. (**D**) The EYFP expression in released sporocyst. Bar = 5 μm. (**E**–**H**) Validation of EYFP location by immunoelectron microscopy (IEM). EYFP was stained in stieda body (black arrow) and the cavity within the sporocyst (white arrow). (**G**) and (**H**) are zoomed-in from (**E**) and (**F**), respectively. IEM was conducted with rabbit anti-EYFP polyclonal antibodies and goat anti-rabbit IgG conjugated to 10-nm gold particles. Bar = 1 μm (**E**); Bar = 500 nm (**F**–**H**).

The success of stable transfection opens up genetic manipulation of *E. acervulina*^23^. For enhanced manipulation, several promoters and signal peptides were screened for using in *E. acervulina*. We tried to clone promoters and signal sequences of 9 candidate genes from *E. acervulina*, including microneme adhesive repeat domain containing protein (EaMCP1), EaMIC1, EaMIC2 and 6 rhoptry proteins (EaROP0, EaROP2, EaROP17, EaROP23, EaROP30, EaROP35) (Table 1). Then we constructed EYFP-expressing plasmids containing each of these elements and conducted transfection in *E. acervulina* sporozoites (Fig. 3A, M/M/R represents one of the above 9 genes). Finally, we obtained EYFP positive progeny oocysts from transfection with a plasmid containing EaMCP1 or EaMIC2 promoters and signal sequences, respectively. The results suggested that the EYFP expression profile was similar in these 2 transfectants, which seemed in the cavity within the sporocyst in the sporulated oocysts (Fig. 3B,C). The location was confirmed by fluorescence microscopy of sporocyst (Fig. 3D), and immunoelectron microscopy (Fig. 3E–H).

**Table 1:** Promoter and signal sequences tested for controlling EYFP expression in *E. acervulina.*

Gene ID	Protein name	Predicted ss	Constructs	EYFP expression
EAH_00017570	MCP1	√	√	√
EAH_00041150	MIC1	√	√	×
EAH_00000090	MIC2	√	√	√
EAH_00057090	ROP0	√	×	×
EAH_00036740	ROP2	√	√	×
EAH_00014170	ROP17	√	√	×
EAH_00014120	ROP23	√	√	×
EAH_00061960	ROP30	√	√	×
EAH_00045380	ROP35	√	√	×

### Targeting proteins to cell surface or microneme in *E. acervulina*

**Figure 4:**
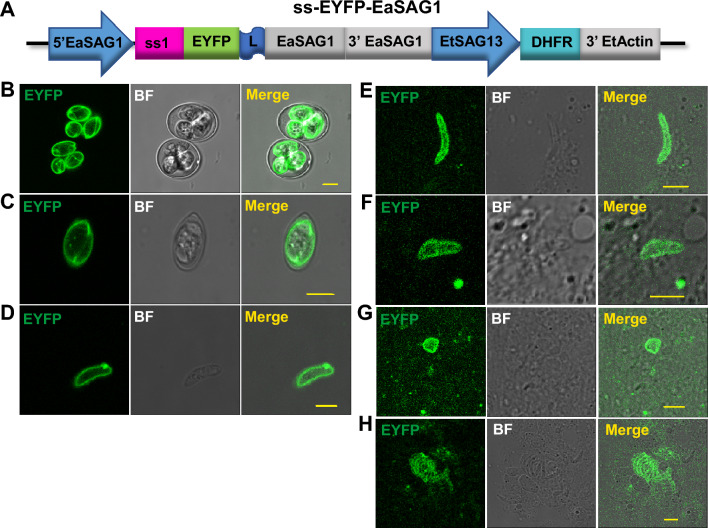
Targeting EYFP to the cell surface of *E. acervulina*. (**A**) The construct ss-EYFP-EaSAG1, in which EYFP is fused between the signal peptide and C terminal mature peptide of EaSAG1. (**B**–**D**) Confocal microscopy for EYFP expression in sporulated oocysts (**B**), sporocysts (**C**), or sporozoites (**D**) mechanically released during oocyst ruptured by shaking with glass beads. Bar = 5 μm. (**E**–**H**) Confocal microscopy for EYFP expression observed during the endogenous developmental stages by duodenum smears. Including non-invaded sporozoites (**E**), invaded sporozoites (**F**), trophozoites (**G**) and schizonts (**H**). Bar = 5 μm. EYFP expresses on the surface of parasite in vivo and in vitro.

In follow-up studies that aimed to validate our strategies for a targeted delivery of cargo proteins, we fused EYFP between the signal peptide and mature peptide of EaSAG for cell surface expression in *E. acervulina* (Fig. 4A). After transfection, we obtained EYFP positive parasites, and the proportion of EYFP positive oocysts in the 2nd generation of progeny was up to 70–80% through double selection of pyrimethamine press and fluorescence-activated cell sorting (FACS). We observed the surface expression of EYFP within sporulated oocysts (Fig. 4B) or released sporocysts (Fig. 4C), or even the sporozoites mechanically released from sporocysts (Fig. 4D). Further, we also observed the surface expression of EYFP during the endogenous developmental stages, including non-invaded sporozoites (Fig. 4E), invaded sporozoites (Fig. 4F), trophozoites (Fig. 4G) and schizonts (Fig. 4H) on slides with duodenum smears. Thus, EYFP mainly concentrated on the parasite surface both in vitro and in vivo, confirming that the in-frame fusion of EaSAG1 between signal peptide and mature peptide could make surface display for heterologous proteins.

**Figure 5:**
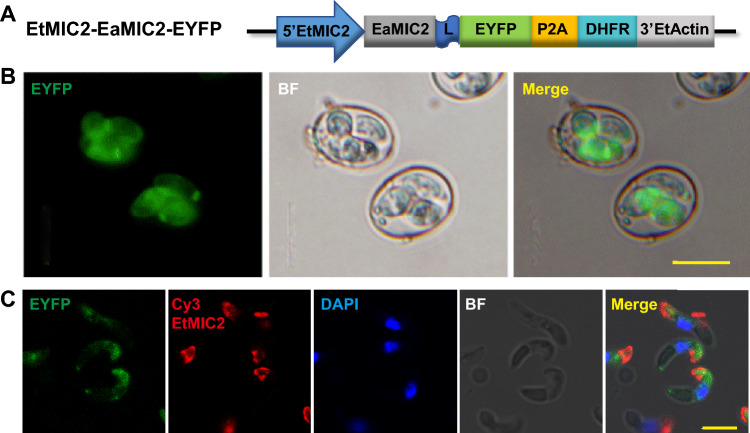
Targeting EYFP to micronemes of *E. acervulina* sporozoites. (**A**) The schematic of plasmid EtMIC2-EaMIC2-EYFP, which was constructed in Fig. 2D. (**B**) Fluorescence microscopy of stably transgenic *E. acervulina*. Bar = 20 μm. (**C**) Immunofluorescent staining for co-localization analysis of MIC2 and EYFP in transgenic sporozoites, using a monoclonal antibody against EtMIC2 and Cy3-conjugated goat anti-mouse IgG (H + L) (Proteintech, America): EYFP expressed in microneme. DAPI, 4',6-diamidino-2-phenylindole dihydrochloride, staining for visualizing the nuclei of the cells. Bar = 5 μm.

For microneme location in *E. acervulina*, we transfected the plasmid EtMIC2-EaMIC2-EYFP (Fig. 2D) in which EYFP was fused to the C-terminal of EaMIC2 under the control of 5’EtMIC2 to *E. acervulina* (Fig. 5A). We obtained EYFP positive parasites, and the proportion of EYFP positive oocysts in the 3rd generation of progeny was 60% with the selection of pyrimethamine and FACS for progeny oocysts (Fig. 5B). We found that the fluorescent signal mainly accumulated in the apical end of EYFP positive sporozoites but fewly co-localized with a monoclonal antibody against the EtMIC2 (Fig. 5C).

## Discussion

Our study here clearly demonstrates that two *Eimeria* species are amenable to genetic modifications that target various cargo proteins (immunogens) to specific location (Table 2). Using confocal fluorescence microscopy, EYFP was observed on cell surface when EYFP is fused between signal peptide and mature peptide of EtSAGs, and in apical end, a potential microneme location when it was fused with MIC2 for both *E. tenella* or *E. acervulina.*

**Table 2:** List of constructs used in the study and their location features.

Constructs	EYFP location	Location sequence	EYFP fusion form
ss13-EYFP-GPI13 (Fig. 1A)	RB	ss13 and GPI13	Between the ss13 and GPI13
ss13-EYFP-EtSAG13 (Fig. 1B)	Microneme and surface (weak)	The whole sequence of EtSAG13	Between the ss13 and the sequence minus ss13
EtSAG1-EYFP (Fig. 1C)	RB	The coding sequence of EtSAG1	C terminus of the sequence
EtSAG1-EYFP-GPI1 (Fig. 1D)	RB	The coding sequence of EtSAG1	Between the sequence minus GPI1 and the GPI1
ss1-EYFP-EtSAG1 (Fig. 1E)	Surface and nucleus	The coding sequence of EtSAG1	Between the ss1 and the sequence minus ss1
EtSAG13-EtMIC2-EYFP (Fig. 2A)	Microneme and nucleus	the coding sequence of EtMIC2	C terminus of the sequence
EtMIC2-EtMIC2-EYFP (Fig. 2B)	Microneme and nucleus	The coding sequence of EtMIC2	C terminus of the sequence
EtSAG13-EaMIC2-EYFP (Fig. 2C)	Microneme	The coding sequence of EaMIC2	C terminus of the sequence
EtMIC2-EaMIC2-EYFP (Figs. 2D and 5A)	Microneme	The coding sequence of EaMIC2	C terminus of the sequence
ss-M/M/R-EYFP (Fig. 3A)	In the cavity within the sporocyst	The ss of EaMIC2 or EaMCP1	C terminus of the sequence
ss-EYFP-EaSAG1 (Fig. 4A)	Surface	The coding sequence of EaSAG1	Between the ss and the sequence minus ss

Various designs that ensure the expression of heterologous proteins intracellularly or extracellularly in transgenic *T. gondii* and *E. tenella* have been described before^6,12,21,24^. These targeting designs always rely on the organelle or surface proteins related to the trafficking mechanism. For surface localization, the C-terminal GPI-anchored protein from TgSAG1 could relocate bacterial alkaline phosphatase^24^ and TgMIC6 to the *T. gondii* tachyzoite surface^25^, which indicate that this sequence contains all the targeting signals required for surface. However, in our research, the GPI from EtSAG1 and EtSAG13 does not lead to EYFP expression on cell surface (Fig. 1A,D), but the whole or coding sequence minus signal peptide does (Fig. 1B,E), suggesting that sequences besides GPI of SAG proteins may also play a role in localization. In addition, when the EtSAG1 was fused to the N terminal of EYFP, EYFP was not shown on surface of sporozoites (Fig. 1C), suggesting that the fusing site relative to the target sequence is an important factor for the translocation of heterologous proteins. There were also puzzling results that EYFP were observed in RBs of extracellular sporozoites (Fig. 1A,C,D). Because there are no studies showing that location sequences of surface protein are related to target proteins to RBs, that are considered to be devoid of a membrane and usually involve in nutrient stores or metabolic energy reserves^26^. This phenomenon may be random because inappropriate fusions with the location sequences of SAGs, but this preference for RBs is worth further study.

For microneme localization, the whole EtMIC2 targeting the foreign protein to the apical end have been reported^13^, which agrees perfectly with our observation: we also targeted EYFP to apical end by fusing EYFP to the C-terminal of EtMIC2 in extracellular sporozoites (Fig. 2A,B), and this position is the ideal location of the EtMIC2^27^. And we showed EaMIC2, which was located mainly at the apical tip of the sporozoite and in the merozoite^28^, also could target EYFP to apical end (Fig. 2C,D). In addition, we showed that the location of MIC2 was not affected by different promoters. The signal peptide of MIC proteins translocated the heterologous proteins to all over the cavity of sporocysts in *E. tenella*^12,13^. In this study, we showed that signal peptide of EaMIC2 and EaMCP1, could target EYFP to the cavity of sporocyst in *E. acervulina* (Fig. 3). And some of the above strategies for surface or microneme could target EYFP to the corresponding position in *E. acervulina* (Figs. 4 and 5). Though EYFP fewly co-localized with a monoclonal antibody against the EtMIC2, it mainly accumulated in the apical end of sporozoites (Fig. 5C), the position where EaMIC2 located^28^. But the colocalization need to be identified in further study. Even so, another species of *Eimeria*, *E. acervulina* was developed further for potential vaccine carrier.

Although we achieved specific targeting of heterologous proteins in two *Eimeria* species, the transport mechanisms are still not fully understood. Our study showed that GPI-anchored sequence is not the only sequence that determines surface location. But the control mechanism of MICs trafficking seems clearer that the signal peptide is responsible for extracellular secretion, and the C-terminal sequence is responsible for targeting to the micronemes^29^. We did not make a further exploration for the specific selected proteins/region for targeting, which should be resolved in the future, especially for understanding the interactions between the *Eimeria* parasites and the host. On the other side, the using of the whole organelle proteins may play a positive role on inducing immune responses for the host, because that they were known as protective antigens^28,30–32^. As research continues with the use of genetically engineered *E. acervulina* parasites as live vaccine vectors, additional data should resolve some of the issues related to mechanisms, as well as assessment of efficacy for vaccine-induced immune responses.

Our studies provide methods for heterologous protein localization in transgenic *Eimeria*, which is the first implementation in *E. acervulina.* These strategies are expected to benefit future studies that use live *Eimeria* parasite as vaccine vectors that induce both cellular and humoral immune responses.

## Methods

### Ethics statement

The animal studies described in this work followed institutional guidelines for animal welfare and biosafety and were approved by China Agricultural University Institutional Animal Care and Use Committee (CAU20160628-2). The study is reported in accordance with the ARRIVE guidelines.

### Parasites and animals

The wild type strain of *E. tenella* (EteWT) used in this study is the *E. tenella* Houghton strain, and the wild type strain of *E. acervulina* (EacWT) is the *E. acervulina* Beijing strain, as maintained in coccidia-free, Arbor Acre (AA) broilers by the National Key Laboratory of Veterinary Public Health and Safety at CAU. Procedures for the collection, purification, sporulation^33^, and extraction of sporocysts and sporozoites were carried out as previously described^34^, except that when detecting EYFP expressing in transgenic *E. acervulina* with surface location, sporulated oocysts in PBS were vortexed in the presence of glass beads until rupturing of most walls of oocysts and sporocysts, sporozoites were observed by fluorescence microscopy, in the mixing state with unbroken oocysts, sporocysts, oocyst shells, and sporocyst shell.

One-day-old AA broiler chickens were purchased from Beijing Arbor Acres Poultry Breeding Co., Ltd. They were fed with a pathogen-free diet and water ad libitum in coccidian-free housing units. After the experiment, the cervical dislocation was performed for chickens necessary for euthanasia, which aims to lose consciousness of chickens rapidly.

### Plasmid construction

Total RNA was extracted from sporozoites of EacWT and EteWT^35^ by using the TRIzol reagent (Invitrogen, USA). cDNA was synthesized through HiScript^®^ III 1st Strand cDNA Synthesis Kit (+ gDNA wiper). The signal sequence, GPI-anchored sequence and/or coding sequence of EtSAG13 (gene ID: ETH_00013178), EtSAG1 (gene ID: ETH_00010835), EaSAG1 (gene ID: EAH_00003690), EtMIC2 (gene ID: ETH_00006930) and EaMIC2 (gene ID: EAH_00000090) were amplified by PCR taking cDNA as the templates.

Genome DNA (gDNA) were prepared from sporulated oocysts of EteWT and EacWT as previously described methods^36^. The whole gene sequence of EtSAG13, promoter sequence and signal sequence of *E. acervulina* microneme adhesive repeat (MAR) domain containing protein 1 (EaMCP1) (gene ID: EAH_00017570, EaMCP1), EaMIC1 (gene ID: EAH_00041150), EaMIC2 (gene ID: EAH_00000090),EaROP0 (gene ID: EAH_00057090), EaROP2 (gene ID: EAH_00036740), EaROP17 (gene ID: EAH_00014170), EaROP23 (gene ID: EAH_00014120), EaROP30 (gene ID: EAH_00061960) and EaROP35 (gene ID: EAH_00045380) were amplified by PCR taking gDNA as the templates.

Signal peptide was predicted online using SignalP (http://www.cbs.dtu.dk/services/SignalP/), and GPI was predicted online using big-PI (Predictorhttp://mendel.imp.ac.at/gpi/gpi_server.html) and referred to the article^37^.

The genes DHFR and EYFP were amplified by PCR using plasmid pSDEP2ARS^38^ as a template. And the backbone was acquired by linearizing the pSDEP2ARS^38^ with SnaBI.

The amplification primers and the information of above products are shown in Supplementary Table S1. These products were cloned into pEASY^®^-Blunt Cloning Vector (TransGen Biotech Co. Ltd, Beijing) for sequencing and extracting plasmids. Then these plasmids were used as templates for fragments needed by final plasmid construction using Seamless Cloning and Assembly kit (TransGen Biotech Co. Ltd, Beijing).

### Transfection and selection of transgenic parasites

The constructed plasmids, linearized with SnaBI, were transfected into 1 × 10^7^ EacWT or EteWT sporozoites by restriction enzyme-mediated integration as previously described^39^. After nucleofection (Program U-033, AMAXA, Switzerland), the transfected *E. tenella* sporozoites were inoculated into the ileocecal opening via the cloaca, and the transfected *E. acervulina* sporozoites were inoculated intravenously into the wing vein of five 1-week-old chickens equally for stable transfection^23^. The transgenic oocysts were selected in chickens by the MoFlo^®^ Cell Sorter (Dako-Cytomation, Fort Collins, CO) on the single-cell mode in vitro, and/or 150 mg/L pyrimethamine (Sigma-Aldrich Co., St. Louis, Mo., USA) press by drinking water in vivo, then inoculated into coccidian-free chickens for the propagation of next generation oocysts.

### Passage in vivo

For *E. tenella*, 1 × 10^4^ oocysts from the 1st generation transgenic population were orally inoculated to an average of five 10-day-old, coccidia-free AA broilers, and the next generation was collected during 6–9 days post inoculation under the drug press. For *E. acervulina*, 6 × 10^4^ oocysts from the 1st generation transgenic population were orally inoculated to an average of three 7-day-old, coccidia-free AA broilers, and the next generation was collected during 5–8 days post inoculation under the drug press.

### Observation of fluorescent reporter protein

Micrographs of the transgenic populations, including sporulated oocysts, sporocysts, or sporozoites expressing EYFP was observed using the fluorescent microscope (OLYMPUS, Japan) and/or confocal microscope (SP5, Leica, Germany).

Eight 7-day-old AA broilers were orally inoculated with 1 × 10^6^ sporulated oocysts of transgenic *E. acervulina.* Every 12 h from 24 to 108 h post inoculation, one chicken was euthanized and necropsied to collect the middle part of duodenum with 3 cm, which was washed with cold PBS, then the mucosa was scraped for smears and observed for endogenous developmental stages using confocal microscope.

For in vitro observation, 2 × 10^5^ freshly purified transgenic sporozoites were inoculated onto human foreskin fibroblast (HFF) monolayers grown on glass coverslips in 12-well plates. Then indirect immunofluorescence assay was conducted 12 h post invasion as previously described^23^, with a monoclonal antibody against EtMIC2 and Cy3-conjugated goat anti-mouse IgG (H + L) (Proteintech, America). 5 × 10^6^ freshly purified transgenic sporozoites were fixed in 2% glutaraldehyde for 24 h, following a routine concluding washing, dehydrating, embedding and sectioning, immunoelectron microscopy assay was conducted as previously described^12^.

### Supplementary Information

Supplementary Table S1.

## Data Availability

All data and materials are present in the contents of this manuscript.

## Abbreviations

GPI1: Glycosylphosphatidylinositol (GPI)-anchored sequence of EtSAG1
GPI13: GPI-anchored sequence of EtSAG13
SS1: Signal sequence of EtSAG1
SS13: Signal sequence of EtSAG13
